# Mr. Bradley's *Case of Extensive Burn*

**Published:** 1813-03

**Authors:** J. A. Bradley

**Affiliations:** Surgeon, Member of the Royal College of Surgeons, London. Liverpool


*To the Editors of the Medical and Physical Journal.*

GENTLEMEN,

IF you think the following case of burn, treated agreeably to Dr. Kentish's method, worthy of a place in your ex-

 tensivelytensively-circulated Journal, you will favor me by inserting it. I am, Gentlemen,

Your obedient Servant,

J. A. BRADLEY, Surgeon,

Member of the Royal College of Surgeons, London.

*Liverpool, Jan.* 9, 1813.

Mary Taylor, aged seven years, (Monday, Sept. 14, 1812,) was dreadfully burnt by her clothes catching fire. Three large and deep eschars occupied the whole of the left side of the back, from the shoulder to the nates; another, equally deep, extended from the top of the left shoulder to the bend of the arm. I arrived at the house about half an hour after the accident had happened, and immediately applied the warm Ol. Terebinthinæ, after the manner recommended by Dr. Kentish. The little patient being put to bed, the Liniment. Terebinth. was spread thick upon old linen, and immediately applied to the injured surfaces. Upon leaving the house, I requested two drops of Tinct. Opii to be given in a glass of warm wine and water.

Tuesday, 15th. Visited her about ten o'clock a. m. and found her pretty free from pain. She had had a little sleep during the night; pulse quick; tongue furred; prescribed 12 grs. pulv. Jalapii, with 2 of calomel, to be taken directly in a little treacle. The Liniment. Terebinthinæ to be continued.

Wednesday, 16th. Visited at eleven o'clock a. m. Suppuration established; the powder had purged twice; fever considerably abated; had slept soundly all night. The Liniment. Terebinthinæ to be discontinued, and the Ung. e Lap. Cal. to be substituted in its place.

Thursday, 17th. Suppuration going on kindly; bowels costive; prescribed the powder as before, to be taken directly; but, as it had not remained upon the stomach, gave a bolus with 4 grs. of calomel at bed-time.

Friday, 18th. Found her free from fever; the bolus had operated well; slept soundly during the night, but great pain this morning of the arm. Prescribed ten drops of Tinct. Opii. Dressings as before.

Saturday, 19th. Profuse suppuration; sloughs separating; appetite good; free from fever.

Sunday, 20th. Visited at ten o'clock a. m.; found the sloughs throwing off, leaving deep ulcerations, particularly in the arm. Continue the Ung. e Lap. Cal.

Monday, 21st. Suppuration lessened, and the parts looking healthy. Dressings as before.

Tuesday, 22d. Tongue furred; pulse quick; skin very hot hot and dry; prescribed a table-spoonful of Aq. Ammon. Acet. to be taken thrice a-day.

Wednesday, 23d. Pulse low; free from fever; the parts put on a healing appearance; dressings as before.

Thursday, 24th. Every thing going on favorably, except a very profuse secretion of matter. Directed the cavities of the sores to be filled with the Pulvis Cretæ, and the bowels to be purged, to lessen this profuse secretion of pus. Dressings as before.

Friday, 25th. The parts healing; the secretion of pus considerably lessened; appetite good; and bowels in good order.

Monday, 28th. Visited again. Found the granulations luxuriantly elevating themselves above the level of the surrounding skin. I tried the effects of Mr. Baynton's plaister-bandage, applying it rather tightly, in strips of about an inch broad, to repress this hypersarcosis.

Tuesday, 29th. Found the plaister-bandage had produced a wonderful effect in lessening the circumference of the sores, but not so much in repressing this exuberant flesh as I expected.

Wednesday, 30th. Ulcers healing rapidly, requiring, occasionally only, the application of caustic, to keep down this hypersarcosis. By the continuance of the same treatment, my little patient, in a few days more, was completely well.

Thus was this dreadful burn cured by the above mode of treatment in as few days, I am convinced, as it would have taken weeks, by the old, vague, and inefficient, treatment, still, I am sorry to say, pursued by many.

*Remarks.*—It is not on account of any thing intrinsically valuable in this case, that I have been induced to draw it up for the pages of this Journal, but because it may, I hope, excite some of your correspondents to give to the younger part of the profession their farther and more matured experience on the subject; and because I have seen, with considerable regret, that the old, vague, and inefficient, mode of treating this affection is still in vogue, notwithstanding the enlightened and philosophic manner in which the subject has been treated by Dr. Kentish himself, as well as by the different gentlemen who have contributed to the support of your inestimable periodical production.

If this obsolete practice had been confined to a few of those petits maitres whose practice is chiefly made up of what they have aped of their superiors, I should not have been so much astonished; but, when we observe it still pursued by those practitioners whose situations in life ought to have taught them a better lesson, it then becomes matter of

 reprehension.

reprehension. Even some of our public hospitals form no exception to these remarks.

The author of an admired modern system of surgery, when speaking of Dr. Kentish's theory, expresses himself no better than thus: "The reasoning seems repugnant to all the received doctrines of inflammation, and, in my mind, is illogical."

That the Servum Pecus should have expressed themselves thus, would not be a thing at all to be wondered at:— "O imitatores, servum pecus; ut mihi sæpe bilem, sæpe jocum vestri imovêre tumultus."

That such characters as these, I say, should frame such an objection, is not at all astonishing; but that the author of the first lines of the Practice of Surgery should give into such reasoning, shocks all the grounds of human belief.

If the gentleman means, by illogical, that which is contrary to the old-school system of inquiry, he may be correct, in as far as this was almost invariably the converse of truth; but if, on the contrary, he means by illogical that which is irrational; then, I think, it will be generally admitted that his opinion, on this subject, is not entitled to that deference which in other matters we are inclined to pay him. In my opinion, the practice recommended, and so very ably enforced, by Dr. K. is highly philosophical, and agreeable to many of the analogies of nature.

If, when the living principle has been nearly destroyed by an intense degree of cold, we are directed, even by Mr. Cooper himself, to apply the accustomed heat in a cautious and gradual manner; can it be illogical, when the same principle has suffered by a high degree of heat, to apply cold with the same precautions? If the stomach had been injured by the stimulus of ardent spirits, would it be illogical to decrease, not suddenly, but gradatim, this powerful irritant?

It will be seen that the above case was treated agreeably to Dr. K.'s method, with the exception of the plaister-bandage, after the inflammation had subsided, and the ulcers reduced to that indolent and fungus state which so generally follows burns.

The application of the plaister-bandage I first learnt from Mr. Simmons, senior surgeon of the Manchester Infirmary, whose successful mode of treating every species of ulcer, with this simple but efficacious dressing, has often excited my admiration.

